# Tributyltin and Vascular Dysfunction: The Role of Oxidative Stress

**DOI:** 10.3389/fendo.2018.00354

**Published:** 2018-07-12

**Authors:** Karoline de Sousa Ronconi, Ivanita Stefanon, Rogerio F. Ribeiro Junior

**Affiliations:** ^1^Department of Physiological Sciences, Federal University of Espírito Santo, Vitória, Brazil; ^2^Department of Pharmacology, University of California, Davis, Davis, CA, United States

**Keywords:** organotin compounds, tributyltin, vascular dysfunction, NADPH oxidase, endothelial dysfunction, nitric oxide

## Abstract

The organotin compounds (OT) are used as fungicides, stabilizers in plastics, miticides, manufacturing and agricultural biocides, wood preservatives and antifouling agents. Tributyltin (TBT) is an OT that was first used for antifouling because it was the most effective agent to prevent undesirable accumulation of marine organisms on solid surfaces, such as ships' hulls or mechanical components, immersed in saltwater. TBT can be easily absorbed by mammals through ingestion, and its cytotoxic effects have become a major concern since their discovery in the 1970s. Recently, it has been demonstrated that TBT exposure is detrimental to the cardiovascular system. TBT is a membrane active substance and its action seems to depend on the OT lipophilicity. As a result, TBT crosses the cell membrane and damages the endothelium and the smooth muscle cells. TBT exposure induces vascular dysfunction, most likely due to endothelial dysfunction and morphological changes in the vascular wall. In an experimental rodent model, small doses of TBT (100 and 500 ng/kg/bw/day for 15 days) modified the vascular reactivity in aorta, mesenteric and coronary arteries followed by smooth muscle cell atrophy, increased collagen deposition and fibrin accumulation. TBT exposure increases oxidative stress by inducing vascular superoxide anion production derived from NADPH oxidase and decreases nitric oxide (NO) production as well as eNOS protein expression. The goal of this review is to summarize the current state of the art regarding the mechanisms involved in the vascular and endothelial dysfunction induced by TBT.

## Introduction

The organotin (OT) compounds have covalent bonds between tin (Sn) and carbon (C). Organotins are used as fungicides, as stabilizers in plastics, molluscicides, miticides, manufacturing catalysts, industrial and farming biocides, wood preservatives and antifouling agents (1–3). Tributyltin (TBT) was first used as an antifouling substance in the early 1960s and came to be recognized as the most effective agent used to prevent accumulation of aquatic organisms on solid shells, such as ships' hulls or motorized components, immersed in seawater. Over a decade ago, TBT copolymer (TBT-SPC) paints probably covered 70–80% of the world's fleet, leading to important economic benefits (4). Twenty years after TBT paints were introduced, it was demonstrated that they are deleterious to aquatic organisms. Even very low concentrations, such as 1 ng/L, were sufficient to cause imposex, as seen in noncommercial *Nucella lapillus* populations around Scottish oil ports, and along the south coast of England (5). Concentrations above 2 ng/L inhibited proper calcification of the commercial oyster, *Crassostrea gigas*, and concentrations above 20 ng/L inhibited larval growth (6).

Considering the harmful effects of TBT compounds on aquatic organisms, restrictions on use were imposed after the 1980s. The International Maritime Organization (IMO) approved a global prohibition on the use of TBT-based antifouling paints (4). However, countries lacking controlling national or regional legislation continue to use organotin compounds (OT) in coast-to-coast routes, mainly due to the lack of equivalent substitutes (4). In addition, TBT can be detected in marine biota and residue 20 years after initial contamination, due mainly to its high lipid-solubility. As a result, TBT residues can be found in organisms throughout the food chain, including mollusks, fish, seabirds and marine mammals (7, 8).

Humans are often exposed to TBT by the ingestion of contaminated seafood, water and beverages. TBT concentrations can vary in marine foods, so it is expected that different diets may cause different concentrations in human tissues and blood (9–11). Based on immune function studies, the World Health Organization adopted an Acceptable Daily Intake value for TBT of 250 ng/kg/day (4). However, due to uncertainty in human-rat toxicity extrapolation, a safety factor of one hundred was used for the final calculation of the daily intake value. The concentrations of TBT in human blood range from 20 to 50 ng/L in males and 170 to 670 ng/L in females and a study that analyzed blood samples from 38 volunteers from Michigan (USA) showed TBT concentrations ranging from below the detection limit up to 1,550 ng/L (12).

It is not clear in the literature what the TBT concentrations in the population are. There is a lack of clinical studies showing the TBT concentrations in human blood and tissues. Furthermore, as TBT can be easily absorbed by mammals, TBT cytotoxicity became a major concern since the discovery of the toxic effects in the 1970s. Consequently, investigators sought to better understand the impact of TBT pollution on the organism. In recent years, more focus has been put on the effects of TBT on the cardiovascular system. In addition, new evidence in the literature demonstrates that TBT exposures of 0.1–0.5 μg/kg/day, at or below the established Acceptable Daily Intake, are detrimental to the cardiovascular system (13–15).

The goal of this review is to summarize the current state of the art regarding TBT and vascular dysfunction, focusing on the mechanisms involved.

## The mechanisms which by organotin induces vascular dysfunction: the role of Nox and oxidative stress

Vascular endothelial homeostasis is a tight balance between vasodilatation and vasoconstriction, pro-thrombotic, pro-inflammatory and anti-thrombotic, anti-inflammatory processes. Endothelial dysfunction can be defined as a shift of the endothelium toward reduced vasodilation, followed by increased vasoconstriction, increased platelet aggregation and adhesion leading to a pro-thrombotic state, enhanced smooth muscle proliferation, and increased vascular inflammation. Endothelial dysfunction is also characterized by reduced activity of key vasodilators such as NO; prostacyclin and EDHF are also recognized as vasodilators. On the other hand, reactive oxygen species (ROS) like the superoxide anion (${\text{O}}_{2}^{-}$), peroxynitrite (ONOO^−^), as well as endothelin-1, thromboxane A as well as angiotensin II are potent vasoconstrictors (16).

**Figure 1:**
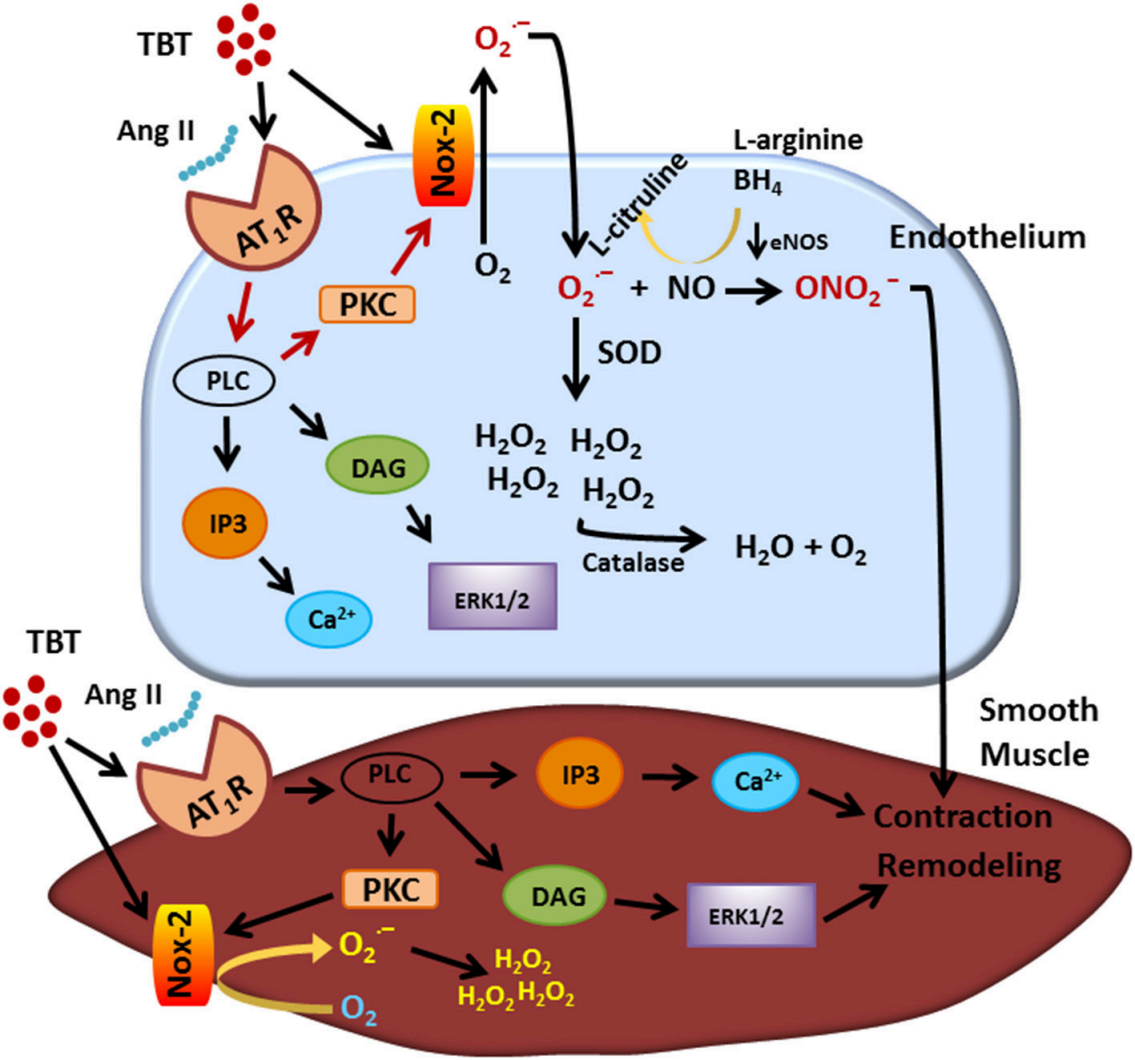
Visual abstract showing the main actions of TBT on endothelial and smooth muscle cell. TBT, tributyltin.

In recent years, vascular dysfunction attributed to TBT exposure has been thought to be manifest mainly in the above-described endothelial changes and also morphological changes in the vascular wall. Figure 1 shows a summary of the main actions of TBT on the endothelium and on the smooth muscle cell. The endothelium is organized in a single layer of cells that are in direct contact with plasma, making these cells vulnerable to the molecules and ions there. As TBT is very lipophilic, it can easily cross the cell membrane and damage the endothelium as well as the smooth muscle cells.

In an experimental rodent model, a small dose of TBT (500 ng/kg) was shown to modify the vascular reactivity, increasing the vasoconstrictive response to phenylephrine in the aorta and in mesenteric arteries (13, 14), while an even smaller dose (100 ng/kg) of TBT decreased the vascular reactivity to phenylephrine in rat aorta (15). Specific TBT effects on vascular reactivity thus depend both upon its concentration and on the particular vascular bed examined.

Furthermore, manifestations of endothelial function have been demonstrated at both high and low TBT doses, in conductance and resistance arteries. Rodrigues et al. (15) showed that low dose TBT exposure (100 ng/Kg/day for 15 days) in female rats induced aortic atrophy, reduced wall thickness and reduced aortic wall surface area. The reduced vasoconstrictor response to phenylephrine, described just above, was characterized by an imbalance in NO bioavailability and an increase in ROS production. Although the authors did not validate this with a specific Nox inhibitor, their evidence suggests that NADPH oxidase is involved in the vascular dysfunction induced by TBT.

The same low dose (100 ng/kg) of TBT was able to induce vascular dysfunction in the coronary arteries in isolated rat heart (17). These hearts presented elevated interstitial collagen deposition, increased coronary pressure and decreased estradiol-induced vasodilation. The authors demonstrated that TBT induced endothelium denudation and platelet aggregation.

The toxicity of TBT was also demonstrated in cultured porcine aortic endothelial cells (18). TBT influenced the expression of markers involved in endothelial cell structure and function, indicating that TBT altered endothelial cells' shape, disrupted their assembly and interfered with their capability to interact with other cells (19). TBT also desensitized dose-dependent ANP-induced relaxation in isolated aortic rings of rats (20).

Ximenes et al. (13) exposed rats for 15 days to a TBT dose (500 ng/kg) that was larger but yet close to the Acceptable Daily Intake, and showed abnormalities in isolated aortic rings characterized by increased vasoconstriction to phenylephrine and KCl. TBT also decreased acetylcholine- and sodium nitroprusside-induced vasorelaxation, and increased oxidative stress. It seems that exposing rats to TBT increases superoxide anion production and hydrogen peroxide, for which the main sources are NADPH oxidase and xanthine oxidase, respectively. Similar to the results in ref. Rodrigues et al. (15), these authors also demonstrated that animals exposed to TBT presented aortic atrophy, increased collagen deposition and fibrin accumulation.

**Figure 2:**
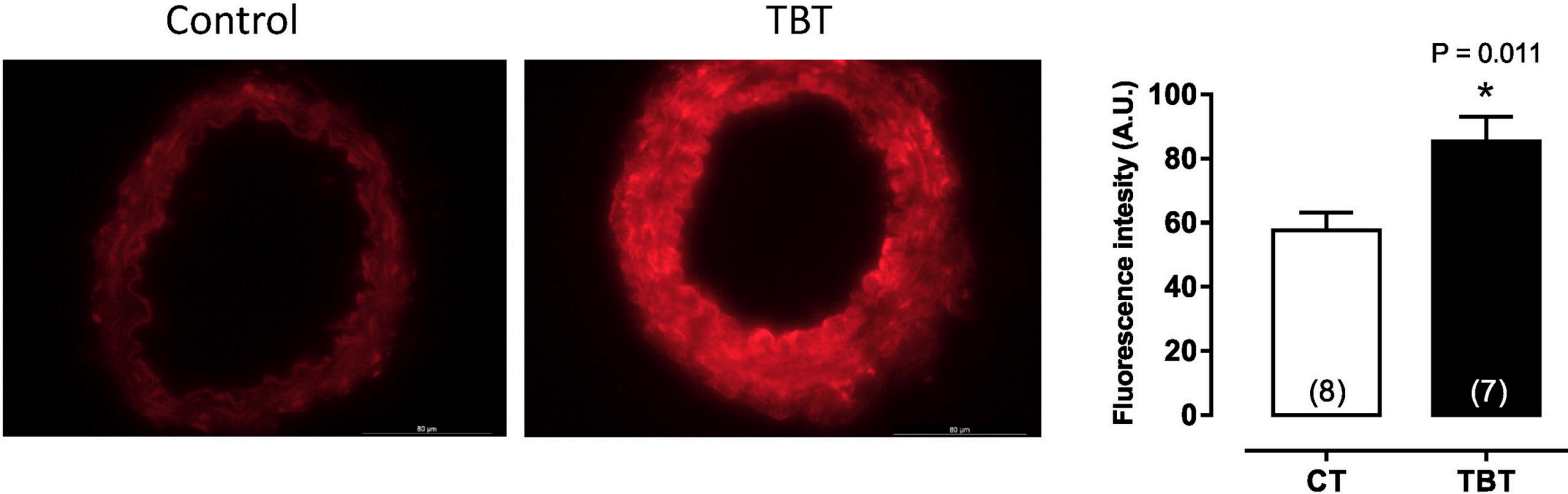
Tributyltin increases vascular superoxide anion production in mesenteric arteries. Animals were treated with TBT (500 ng/Kg) for15 days. This figure is reprinted from Ribeiro Junior et al. (14).

This same dose of TBT has been shown to cause structural and mechanical abnormalities in mesenteric arteries. Resistance arteries have a central role in the maintenance of blood pressure and tissue perfusion, roles that are dependent on the capability of smooth muscle cells to contract and relax to vasoactive components (21, 22). Ribeiro Junior et al. (14), demonstrated that mesenteric arteries of rats treated with TBT (500 ng/kg) for 15 days showed increased phenylephrine-induced vasoconstriction and morpho-functional abnormalities. As shown in Figure 2, TBT exposure increased superoxide anion production derived from NADPH oxidase. It also decreased NO production as well as eNOS protein expression.

The vascular abnormalities induced by TBT in the mesenteric smooth muscle cells could involve angiotensin II receptor and gp91phox pathways. The increased artery collagen deposition could contribute to the enhanced vascular stiffness and increased pulse wave velocity in TBT-treated animals. Using Nox specific inhibitors, the authors characterized how NADPH oxidase induces vascular dysfunction in TBT-treated rats. Both VAS2870 and ML-171 decreased the vascular reactivity in mesenteric arteries from TBT-treated rats. The same response was not observed in control arteries. The authors also showed increased protein expression of Nox2, AT_1_ receptor and ERK 1/2. It seems that TBT enhanced the angiotensin II downstream signaling pathway, leading to inward remodeling and vascular dysfunction. Oxidative stress is an important mediator of vascular remodeling in many vascular beds such as mesenteric and subcutaneous arterioles (23). Chan et al. used Nox-2 knockout animals to demonstrate that superoxide anion generated from the Nox2 isoform oxidase plays a main role in AngII-induced cerebral arteriolar inward remodeling (24).

The Nox family proteins are membrane-bound and they transfer electrons from NADPH to oxygen, generating superoxide anions (25). Among the seven members of the Nox family, Nox-1, Nox-2, Nox-4, and Nox-5 are expressed in vascular human tissue and are involved in regulation of vascular contractility (26, 27). The Nox enzymes are well known to be main players in mediating vascular dysfunction and disease (28–30). The Nox2 oxidase complex is widely distributed in lung, heart and vasculature (31). In addition, angiotensin II receptor activation further stimulates downstream PKC or Rho kinase pathways, leading to Nox-2 activation and superoxide anion production in smooth muscle cells (32–35), contributing to vascular dysfunction.

The mechanisms involving TBT adverse effects on the vascular system need to be better understood, toward eliciting vascular risks associated with even very low concentrations of TBT. According to the Acceptable Daily Intake value for TBT of 250 ng/kg/day adopted by the World Health Organization, doses below 250 ng/kg/day are tolerable. However, the literature we have reviewed here shows that even low doses of TBT induce vascular dysfunction in rodents. To date, effects of acute TBT exposure on vascular function and on cardiac muscle cells remain unexplored. Overall, data in literature are scarce and new studies are needed to understand how TBT affects the cardiovascular system. It is unquestionable that TBT is a risk factor for cardiovascular diseases.

## Author contributions

KR, IS, and RR wrote the general structure of the text, KR, IS, and RR did the literature review, and designed the figures. RR read the final revision of the text.

### Conflict of interest statement

The authors declare that the research was conducted in the absence of any commercial or financial relationships that could be construed as a potential conflict of interest.
